# Hydrogen-Assisted Calcination of CaCO_3_: Kinetics and Mechanistic Insights under Desorption-Enhanced Reverse Water Gas Shift Conditions

**DOI:** 10.1021/acsomega.6c05266

**Published:** 2026-07-21

**Authors:** Gemma Grasa, Yusbeli García, Claudia Navarro, Sergio Celiméndiz, Isabel Martínez, Ramón Murillo

**Affiliations:** Consejo Superior Investigaciones Científicas, Instituto de Carboquímica, C/Miguel Luesma Castán 4, 50018 Zaragoza, Spain

## Abstract

This study investigates the kinetics of CaCO_3_ calcination in H_2_ under conditions relevant to the desorption-enhanced reverse water–gas shift (DERWGS) process. Thermogravimetric experiments performed with natural limestone between 600 and 800 °C showed that H_2_ markedly accelerates calcination, enabling decomposition at temperatures about 150 °C lower than in conventional calcination environments. As a consequence, the CaO obtained under DERWGS conditions exhibited significantly improved cyclic performance, with nearly double the residual CO_2_ carrying capacity of sorbents calcined under standard Ca-looping conditions. For small particles, CaCO_3_ conversion followed a homogeneous pattern and was well described by an Avrami–Erofeev kinetic expression with *n* = 2. The derived kinetic model, which accounts for temperature, H_2_ partial pressure, and the thermodynamic driving force associated with the equilibrium CO_2_ partial pressure, accurately reproduced conversion curves obtained at different temperatures, calcination cycles, and gas compositions. The apparent activation energy was 184 ± 4 kJ mol^–1^, consistent with literature values for reductive calcination. CO_2_ was the only gas-phase species found to directly inhibit calcination, whereas CO and H_2_O had no measurable independent effect. These findings support a sequential calcination–RWGS mechanism and provide a kinetic basis for the design and scale-up of DERWGS systems for low-carbon lime production and CO_2_ utilization.

## Graphical abstract

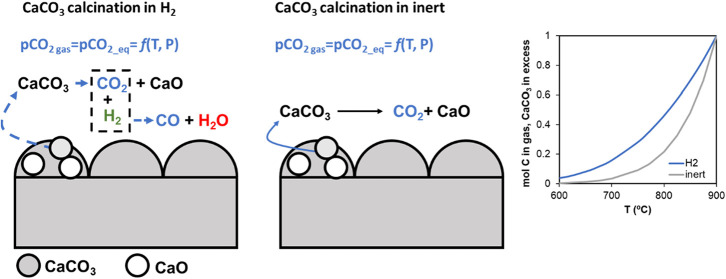

## 1 Introduction

The industrial sector accounts for roughly one-quarter of global energy-related CO_2_ emissions, and technologies such as hydrogen use, direct electrification, and carbon capture, utilization, and storage (CCUS) are expected to contribute to nearly half of the required emission reductions in heavy industries by 2050, according to the Net Zero Emissions by 2050 Scenario.^1^ These technologies are also essential for mitigating process-related emissions arising from inherent CO_2_-generating reactions, such as carbonate calcination during clinker and lime production.^2^ Calcination requires high-temperature heat transfer (above 900 °C), typically supplied by fuel combustion, with energy demands of approximately 3.2 GJ per tonne of lime produced. Despite significant improvements in kiln efficiency, around 69% of CO_2_ emissions from the lime industry remain unavoidable because they originate from the decomposition of carbonates. Owing to lime’s widespread use as a flux, sorbent, and chemical intermediate in sectors including cement and concrete, iron and steel, chemical manufacturing, environmental technologies, pulp and paper, and agriculture, decarbonising lime production offers the potential to deliver substantial indirect emission reductions across multiple hard-to-abate industrial value chains. Consequently, the deployment of CCS is widely regarded as the preferred mitigation pathway for managing large volumes of concentrated CO_2_ emitted by these heavy-emitting sectors. In parallel, however, the utilization of CO_2_ for synthetic fuel and chemical production is gaining increasing attention, driven by the expanding availability of low-cost renewable hydrogen from water electrolysis, which enables CO_2_ conversion routes to complement geological storage in integrated decarbonisation strategies.^3^ Current research efforts therefore focus on developing processes capable of producing concentrated CO_2_ streams, including oxy-fuel combustion, indirectly heated calciners, and end-of-pipe capture technologies such as cryogenic separation, membranes, and absorption systems.^2^

Conventional synthetic fuel production typically relies on a syngas stream containing CO, CO_2_, and H_2_, which must meet stringent compositional and purity requirements prior to downstream catalytic synthesis, thereby necessitating multiple gas conditioning and separation steps that increase process complexity and cost.^4,5^ In the context of CCU applied to hard-to-abate industries such as lime and cement production, these requirements can be particularly restrictive, as CO_2_ is commonly available either diluted in flue gases or mixed with inert gases and trace contaminants. An alternative approach that has recently attracted growing interest is the hydrogenation of metal carbonates, also referred to as reductive calcination.^6–8^ In this concept, a reducing agent—most notably hydrogen—is introduced during the thermal decomposition of CaCO_3_, enabling simultaneous calcination and in situ CO_2_ conversion within a single reactor. Beyond its application to primary lime production, reductive calcination can also be envisaged as an integrated reaction pathway within calcium looping (CaL)-based CO_2_ capture systems, where CaCO_3_ regeneration is a recurring step. By coupling carbonate regeneration with CO_2_ hydrogenation, this approach offers the potential to intensify CaL processes, reduce the need for separate CO_2_ compression and handling units, and directly valorise captured CO_2_ into reduced carbon products. As such, reductive calcination represents a promising bridge between CCS-dominated mitigation strategies and emerging CCU concepts enabled by the increasing availability of renewable hydrogen, while maintaining compatibility with existing lime and CaL-based industrial infrastructures.

Depending on the reducing gas and catalyst employed, reductive calcination can couple CaCO_3_ decomposition with either the reverse water–gas shift (RWGS) reaction (eqs 1–2) or CO_2_ methanation (eqs 1–3),^9^ offering potential benefits in process intensification and energy efficiency.

1 $${\mathrm{CaCO}}_{3}\to \mathrm{CaO}+{\mathrm{CO}}_{2\left(g\right)},{\Delta H}_{298K}=178\;\mathrm{KJ}/\mathrm{mol}$$

2 $${\mathrm{CO}}_{2\left(g\right)}+H_{2\left(g\right)}\to {\mathrm{CO}}_{\left(g\right)}+H_{2}O_{\left(g\right)},{\Delta H}_{298K}=41\;\mathrm{KJ}/\mathrm{mol}$$

3 $${\mathrm{CO}}_{2\left(g\right)}+4H_{2\left(g\right)}\to {\mathrm{CH}}_{4\left(g\right)}+2H_{2}O_{\left(g\right)},{\Delta H}_{298K}=-165\;\mathrm{KJ}/\mathrm{mol}$$

The presence of a reducing atmosphere converts the CO_2_ released from CaCO_3_ into higher-value products such as CO or CH_4_, effectively lowering the CO_2_ partial pressure and shifting the equilibrium in reaction ([Disp-formula eq1]) toward CaCO_3_ decomposition. This strategy has been proposed as a means of reducing calcination temperature^6^ compared with conventional calcination processes designed to deliver a concentrated CO_2_ stream^10,11^ or used in lime and cement manufacture.^2^

Although reductive calcination has received increasing attention in recent years, the majority of existing studies have focused on so-called dual-functional materials (DFMs), in which CaO acts as a CO_2_ sorbent, while an incorporated transition metal provides the catalytic functionality required for either the reverse water–gas shift (RWGS) or methanation reactions.^12–14^ While methane production via reductive calcination inherently requires the presence of an external methanation catalyst,^12^ CaO itself has been shown to exhibit intrinsic catalytic activity for the RWGS reaction, enabling CO formation without the need for additional active metal phases.^15–17^

This manuscript focuses on the combined equilibrium between CaCO_3_ decomposition and the RWGS reaction, a process referred to as the desorption-enhanced reverse water–gas shift (DERWGS), which has only recently been elucidated.^15^ In the DERWGS process, CO_2_ released during calcium carbonate decomposition is simultaneously consumed via RWGS, resulting in a shift in both reaction equilibria and enabling enhanced carbonate conversion under appropriate operating conditions. Experimental studies have demonstrated the feasibility of producing syngas with H_2_/(CO + CO_2_) ratios suitable for downstream synthetic fuel synthesis in packed-bed reactors operated under DERWGS conditions, highlighting the potential of this approach as an integrated pathway for lime production and CO_2_ utilization.

However, further progress in DERWGS reactor development, optimization, and scale-up requires a detailed understanding of the intrinsic reaction kinetics governing both carbonate decomposition and CO_2_ hydrogenation under coupled conditions. While the kinetics of CaCO_3_ calcination have been extensively investigated under inert, vacuum, and CO_2_-rich atmospheres, reflecting conventional lime production and calcium looping operation,^18–23^ kinetic studies conducted under reducing environments remain scarce. This lack of data is particularly critical for DERWGS systems, where the presence of hydrogen fundamentally alters the reaction pathway by coupling CaCO_3_ decomposition with the RWGS reaction. As a result, the applicability of conventional calcination kinetic models to DERWGS conditions is uncertain, highlighting the need for targeted kinetic investigations that can support reactor design, process modeling, and scale-up of integrated lime production and CO_2_ utilization concepts. Reported investigations of reductive calcination include studies performed both in the presence and absence of metal-based catalysts,^9,14,24–26^ covering temperatures in the range of 430–850 °C and hydrogen partial pressures up to 0.1 MPa. Apparent activation energies reported in the literature typically fall between 156 and 236 kJ mol^–1^, with higher values generally observed at lower temperatures and in systems lacking catalytic additives. Shrinking-core-type models have frequently been employed to describe CaCO_3_ conversion behavior under reductive calcination conditions in both fixed-bed and fluidized reactors.^9,25^ Experimental evidence further indicates that the presence of hydrogen can accelerate CaCO_3_ decomposition by up to a factor of 5 compared with inert environments,^25^ while mineral impurities naturally present in limestone have been shown to reduce activation energies relative to those obtained for pure CaCO_3_.^24^ The conceptual process framework considered in this study is illustrated in Figure 1, which depicts the integration of reductive calcination within both once-through lime production systems and cyclic calcium looping (CaL)-based CO_2_ capture configurations. In this scheme, CaCO_3_ regeneration is carried out in a hydrogen-rich environment, coupling carbonate decomposition with in situ CO_2_ hydrogenation via the reverse water–gas shift (RWGS) reaction and enabling the direct production of a syngas-containing stream.

**Figure 1:**
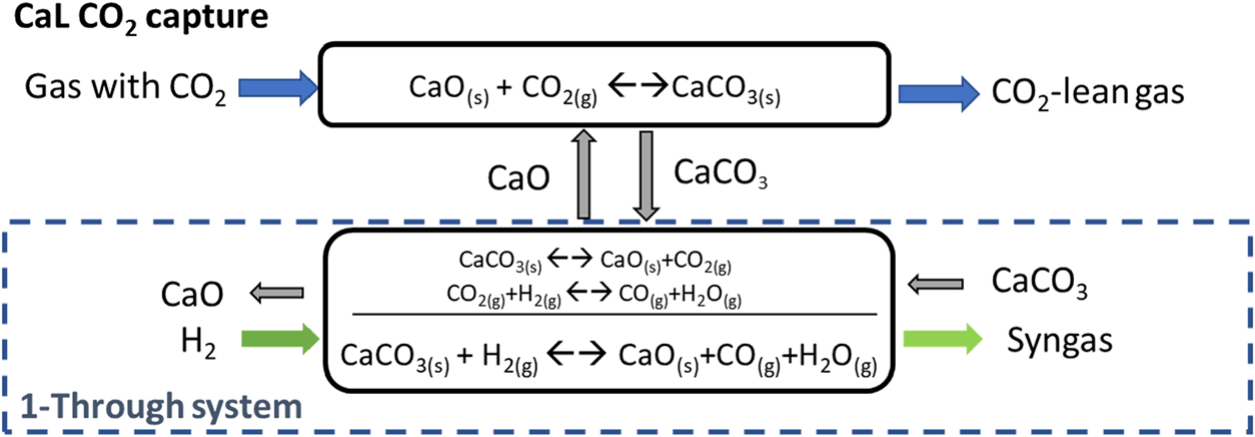
Simplified process scheme illustrating the integration of reductive calcination under H_2_ within conventional calcium looping (CaL) systems and once-through lime production configurations.

The aim of this work is to determine the intrinsic kinetic parameters governing CaCO_3_ decomposition in hydrogen in the absence of additional RWGS catalysts, thereby establishing a fundamental kinetic basis for the design and optimization of reactors operating under desorption-enhanced reverse water–gas shift (DERWGS) conditions. CaCO_3_ conversion to CaO in the presence of H_2_—arising from the coupled effects of calcination and CO_2_ hydrogenation—was investigated under operating conditions selected to maximize CO_2_ conversion to CO, as predicted by DERWGS equilibrium analysis.^15^ The kinetic framework developed in this study is intended to be applicable to both once-through calcination reactors for low-carbon lime production and cyclic CaL configurations, where CaO-based sorbents undergo repeated carbonation–calcination cycles, as schematically represented in Figure 1.

Beyond kinetic parameter estimation,^12,13^ this work seeks to discriminate between competing mechanistic interpretations of reductive calcination through the systematic assessment of gas-phase effects on reaction rates. The literature presents divergent hypotheses, including a direct reductive decomposition of CaCO_3_ to CaO, CO, and H_2_O, and a sequential pathway comprising conventional calcination to CaO and CO_2_ followed by CO_2_ hydrogenation via the reverse water–gas shift (RWGS) reaction.^9,12–14,25^ In the present study, mechanistic insight is inferred indirectly from the kinetic response of CaCO_3_ decomposition to the controlled presence of individual gas-phase species (H_2_, CO_2_, CO, and H_2_O). The central hypothesis is that the observed rate enhancements and inhibitions under DERWGS conditions are consistent with a mechanism dominated by carbonate decomposition coupled with RWGS, rather than by direct reductive cleavage of the carbonate lattice.^15–17^ Establishing this distinction is critical for the development of robust kinetic models applicable to both once-through lime production reactors and cyclic calcium looping (CaL) systems operating under reducing atmospheres.

## 2 Materials and Methods

Natural limestone (90 wt % CaCO_3_, with Si and Mg as the main impurities) was selected as the CaCO_3_ source material for this study. Calcination experiments under H_2_ atmosphere were conducted in an atmospheric thermogravimetric analyzer (TGA). The experimental setup, previously described in detail elsewhere,^27^ is equipped with multiple mass flow controllers for both gases and liquids (N_2_, H_2_, CO, CO_2_, H_2_O, and He). Samples for analysis were placed in a platinum basket inside a quartz reactor, externally heated by a furnace capable of operation up to 975 °C. A series of preliminary calcination tests was first conducted to identify operating conditions that would not influence the determination of intrinsic kinetic parameters. These tests focused on avoiding external and internal mass and heat transfer limitations. Calcination experiments were always carried out under isothermal conditions. The heating ramp to the target temperature was performed under 100 vol % CO_2_, at a rate of 25 °C min^–1^, in order to prevent any decomposition of CaCO_3_ prior to the introduction of H_2_. The temperature was allowed to stabilize before switching the reaction atmosphere. Results from these preliminary tests are included in Figure 2, and based on this analysis, kinetic experiments were performed using a total gas flow rate of 20 lN h^–1^ and approximately 3 mg of sample with a particle-size cut of 100–200 μm (see Figure 2 that contains calcination curves as a function of sample weight, total gas flow, and Figure 5 for particle-size discussion).

**Figure 2:**
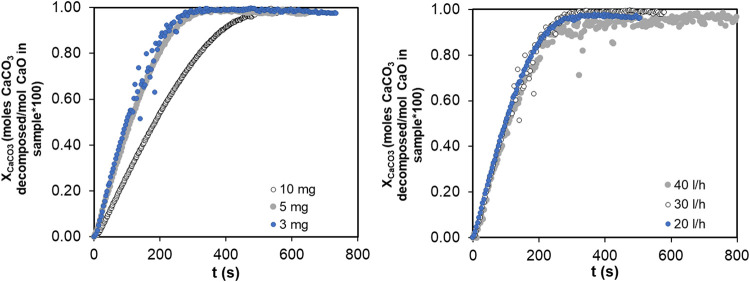
Experimental CaCO_3_ calcination under inert conditions at 750 °C. (Left) For different sample weights and 30 lN/h. (Right) 3 mg sample and different flows.

To determine reaction kinetic parameters, multicycle calcination–carbonation experiments (up to 10 cycles) were then carried out at temperatures between 600 and 800 °C in 100 vol % H_2_. Additional experiments incorporating CO_2_, CO, or H_2_O (balance H_2_) were performed to validate the kinetic parameters across a broader range of gas compositions and to indirectly elucidate the mechanism for the reactions involved. Additionally, the evolution of the CO_2_-sorption carrying capacity along carbonation/calcination cycles in H_2_, some selected tests were extended up to 60 cycles. After each calcination step in H_2_, carbonation was performed at 650 °C for 20 min in a gas mixture containing 20 vol % CO_2_ in N_2_. Fresh material and samples calcined in N_2_ at 850 °C (quartz reactor) were texturally characterized by He pycnometry, Hg porosimetry, and BET surface area analysis. The resulting textural properties are summarized in Table 1.

**Table 1:** Textural Properties of the Limestone and CaO Produced through Calcination at 850 °C in N_2_ Derived from Hg Porosimetry, N_2_ Physisorption and He Picnometry

	density (g/cm^3^)	BET surface area (m^2^/g)	ε
limestone	2.7	<1	<0.05
CaO	3.19	16.8	0.47

## 3 Results and Discussion

CaCO_3_ calcination is an equilibrium-driven reaction, in which the equilibrium CO_2_ partial pressure depends on temperature and total pressure and can be expressed according to eq ([Disp-formula eq4]).^28^

4 $$P_{CO2_{eq}}\left(atm\right)=e^{\left(16.3-19130/T\left(K\right)\right)}$$

When calcination of CaCO_3_ occurs in the presence of H_2_, an additional reaction (RWGS, eq 2) can take place between the H_2_ and the CO_2_ released from the carbonate (or via direct interaction of H_2_ with CaCO_3_), producing CO and therefore promoting CaCO_3_ decomposition. This effect is attributed to the demonstrated catalytic activity of CaO toward the RWGS reaction.^15–17^ As a consequence, the number of carbon moles in the gas phase (as CO and CO_2_) increases relative to CaCO_3_ calcination under inert conditions. The combined thermodynamic equilibrium of CaCO_3_ calcination and RWGS, hereafter referred to as DERWGS, was solved in a recent study.^15^ That work also experimentally demonstrated that, when both reactions occur in the presence of excess CaCO_3_, the CO_2_ partial pressure in the gas phase always remains at the equilibrium value predicted by eq ([Disp-formula eq4]). Under these conditions, the extent of the DERWGS equilibrium is determined by the deviation between the experimentally measured CO concentration and the CO concentration predicted by the combined equilibria. Figure 3 presents the total carbon content in the gas phase as a function of calcination temperature for CaCO_3_ calcination in an inert atmosphere (gray line, calculated using eq ([Disp-formula eq4])) and under H_2_ according to the combined DERWGS equilibrium (blue line).^15^ As shown in the figure, the enhancement of CaCO_3_ calcination induced by RWGS is maximized in the temperature range 650–800 °C.

**Figure 3:**
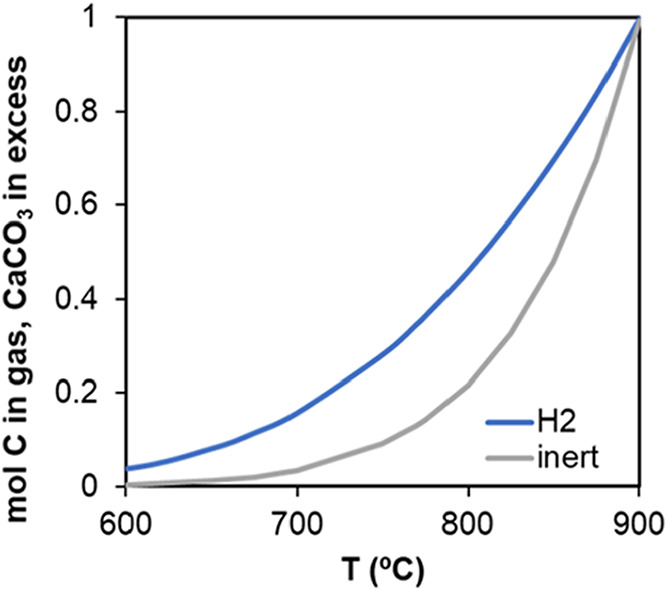
Moles of carbon in the gas phase per mole of gas during CaCO_3_ calcination under inert conditions and under H_2_ according to the DERWGS equilibrium. The gray solid line corresponds to eq ([Disp-formula eq4]), while the blue solid line was calculated from the combined DERWGS equilibrium using the methodology reported by Abanades and Grasa.^15^ The figure is an original representation prepared in this work.

This enhancement was experimentally confirmed by the calcination conversion curves shown in Figure 4 (left), corresponding to CaCO_3_ calcination at 650 °C under inert and H_2_ atmospheres. The results indicate that CaCO_3_ calcination at low temperature under inert conditions is extremely slow, in agreement with previous studies.^19,22^ This behavior is attributed to the low thermodynamic driving force of the reaction, imposed by the equilibrium CO_2_ partial pressure, which at 650 °C and 1 atm total pressure corresponds to 0.0119 atm (eq 4). Consequently, such low temperatures are impractical for industrial processes involving CaCO_3_ calcination under inert atmospheres.

**Figure 4:**
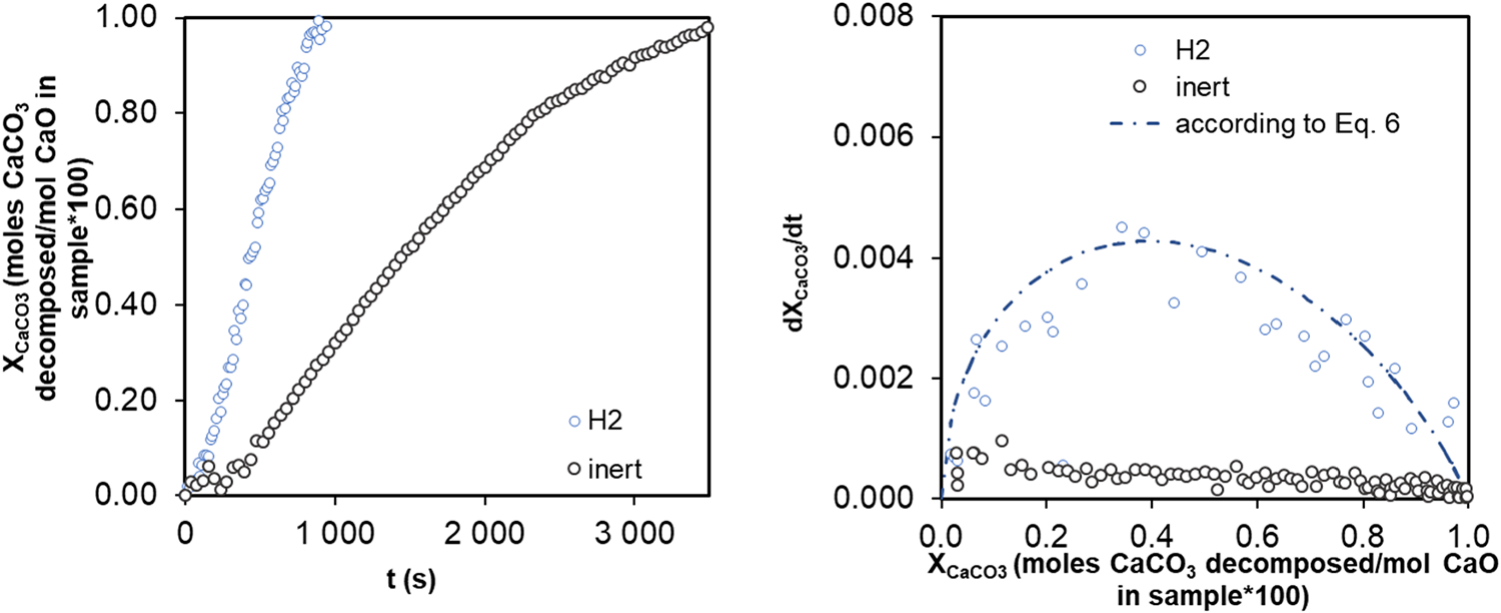
(Left) Experimental CaCO_3_ molar conversion to CaO at 650 °C, under H_2_ and inert atmospheres. (Right) Instantaneous reaction rate as a function of conversion. Dotted line demotes the prediction from eq ([Disp-formula eq6]).

In contrast, when calcination is performed under an H_2_ atmosphere, the reaction rate is markedly accelerated due to the additional driving force provided by the DERWGS mechanism. At 650 °C and 1 atm total pressure, the equilibrium partial pressures of CO and CO_2_ are 0.070 and 0.0119 atm, respectively. The resulting increase in reaction rate (shown in Figure 4, right) is particularly relevant for the design and scale-up of calcination processes operated under reducing conditions, as previous work in packed bed reactors has demonstrated that short solid–gas contact times are sufficient for DERWGS reactions to reach equilibrium and produce syngas with a suitable H_2_/(CO + CO_2_) ratio for downstream synthetic fuel production.^15^

Only a limited number of studies have addressed CaCO_3_ calcination kinetics under reducing conditions (i.e., in the presence of H_2_), and most rely on apparent kinetic models.^9,25^ Given the substantial differences in reaction environment, temperature, and the presence of RWGS reactions that enhance calcination, the generation of reliable kinetic data is essential. Such data are particularly relevant for the scale-up of the DERWGS process and must cover both natural limestone (first calcination) and partially carbonated materials acting as CO_2_ sorbents in Ca-looping (CaL) systems.

To this end, a series of calcination–carbonation experiments was conducted using limestone particles with size ranges of 75–100 μm and 2–3.15 mm at 750 °C under H_2_. Figure 5 shows CaCO_3_ calcination conversion as a function of time for different particle-size cuts and calcination cycles. Figure 5 (left) corresponds to the first calcination of natural limestone, while Figure 5 (right) shows the fifth calcination cycle following four carbonation–calcination repetitions. As observed, increasing particle-size results in a lower calcination rate, suggesting that heat transfer limitations—due to reaction endothermicity—or mass transfer limitations associated with CO_2_, CO, and H_2_O removal may influence reaction progress. Comparison of Figure 5 (left) and (right) reveals that the time required to achieve complete conversion decreases as the number of cycles increases, corroborating earlier findings that calcination time depends on the initial CaCO_3_ content of the material (*X*_carb_).^22^ Furthermore, no significant differences in conversion curves were observed for particle sizes below 200 μm. The influence of particle size on reaction rate also decreased for partially carbonated samples relative to fresh limestone. This behavior can be attributed to the more open pore structure developed after repeated calcination–carbonation cycles that allows representing the evolution of particle conversion through a homogeneous conversion pattern.^22^ Based on these observations, a particle-size range of 100–200 μm was selected for subsequent experiments to determine intrinsic kinetic parameters under conditions free of mass and heat transfer limitations.^22^

**Figure 5:**
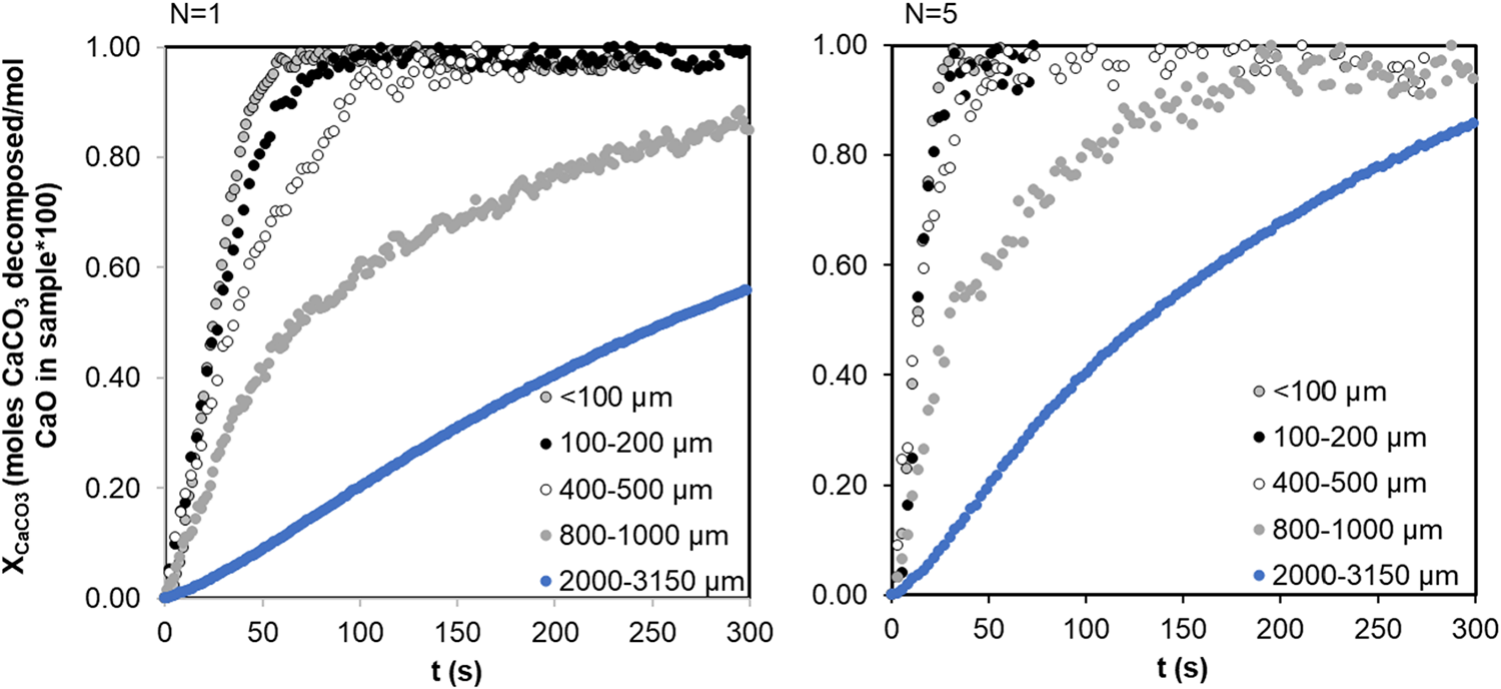
CaCO_3_ conversion to CaO (*X*_CaCO3_) with time at 750 °C under 100% vol. H_2_ for different particle-size cuts. (Left) First calcination of natural limestone. (Right) Fifth calcination (sample partially carbonated, *X*_carb4_ = 0.48).

Focusing on the 100–200 μm particle-size range, Figure 6 (left) presents sample weight loss as a function of time for an increasing number of calcination–carbonation cycles. A clear difference in slope (Δmg/Δ*t*) is observed between the first calcination and subsequent cycles, reflecting the evolution of porosity and pore structure induced by cycling.^22,29^ Figure 6 (right) shows that the time required to reach complete calcination (i.e., *X*_CaCO3_ = 1) decreases with increasing cycle number, consistent with the changes in CO_2_ carrying capacity and reaction rate observed in Figure 6 (left).

**Figure 6:**
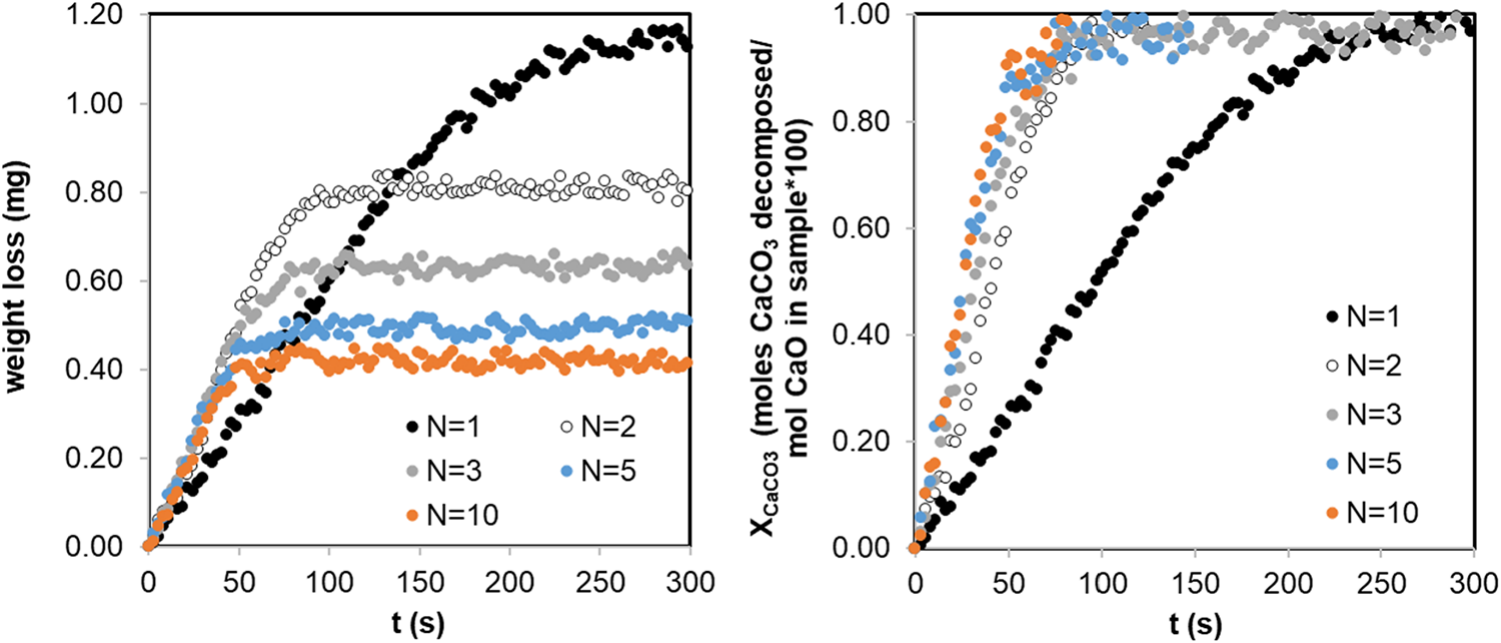
(Left) Sample weight loss with time for an increasing number of reaction cycles at 750 °C and 100% vol. H_2_. (Right) CaCO_3_ conversion to CaO (*X*_CaCO3_) with time for the same test.

Because calcination time depends directly on the CaCO_3_ content of the material, it is essential to evaluate the impact of the proposed calcination conditions—namely temperature, the presence of H_2_, and a low (or nearly zero) CO_2_ partial pressure—on the CO_2_ carrying capacity of the sorbent over multiple calcination–carbonation cycles (*X*_carbN_). These calcination conditions differ substantially from those traditionally reported to promote sorbent sintering and BET surface area loss—typically associated with high temperatures and elevated CO_2_ partial pressures—which ultimately lead to a pronounced decay in CO_2_ carrying capacity.^21,23^

To this end, up to 60 calcination–carbonation cycles were carried out on several samples with a particle-size range of 100–200 μm. Calcination was performed at 750 °C under three different atmospheres: N_2_, H_2_, and H_2_/CO_2_ (with the CO_2_ partial pressure set at 0.5 p_CO2,eq_ at this temperature). Carbonation of CaO was conducted at 650 °C using 15 vol % CO_2_ for 20 min. Figure 7 presents the experimental evolution of CaO carbonation conversion (*X*_carb,N_) as a function of the number of reaction cycles, N, for different calcination atmospheres. The figure also includes the predicted evolution obtained using the semiempirical expression given in eq ([Disp-formula eq5]), previously validated for sorbent decay under calcination conditions aimed at producing a concentrated CO_2_ stream (i.e., calcination at 850–950 °C in N_2_, referred to here as standard conditions).^29^ The equation contains two fitting parameters: k, the decay constant governing the reduction in CO_2_ carrying capacity with cycling, and *X*_r_, the residual carbonation conversion approached at an infinite number of cycles (see Table 2)­

5 $$X_{\mathrm{carbN}}\left(\mathrm{molar}\right)=\frac{1}{\left(\frac{1}{1-X_{r}}\right)+\mathrm{kN}}+X_{r}$$

**Figure 7:**
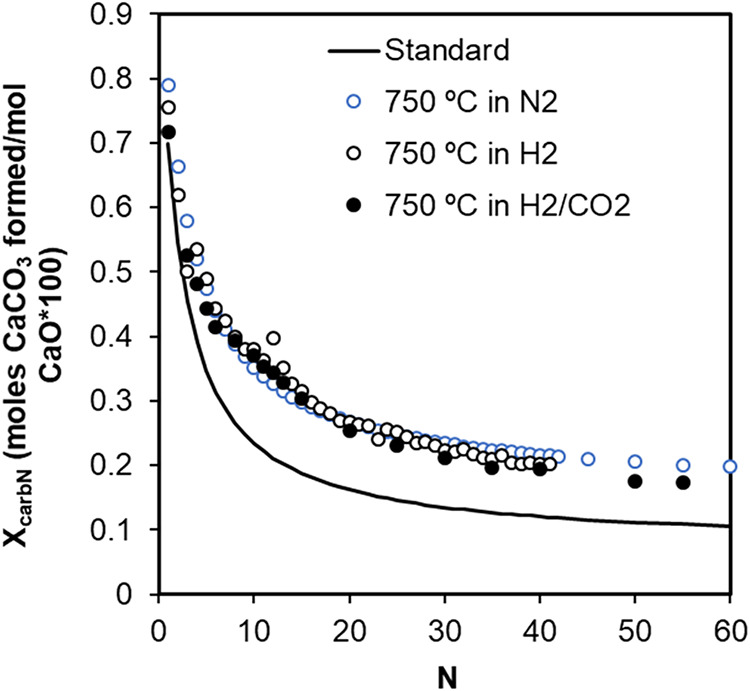
Evolution of CO_2_ carrying capacity (*X*_carbN_) as a function of calcination conditions. CaO carbonation has been performed at 650 °C and 15% vol. CO_2_ for 20 min. Calcination conditions are indicated in the legend, standard means in N_2_ 850–950 °C.

**Table 2:** Constants in Equation ([Disp-formula eq5]) for Different Calcination Conditions

	standard conditions	low-T DERWGS conditions (inert, H_2_, or H_2_/CO_2_ near to equilibrium)
*k*	0.52	0.39
*X*_r_	0.075	0.157

It should be noted, however, that these standard conditions involve significantly higher temperatures than those employed under DERWGS conditions in this work (750 °C). Consequently, the more pronounced decay typically reported in conventional CaL systems is largely associated with enhanced sintering at high temperatures under a rich CO_2_ atmosphere.^21,23^

At the milder temperatures and gas environments explored here (inert, H_2_, and H_2_/CO_2_ near equilibrium), the material exhibits improved stability over cycling with a slower decay in CO_2_ carrying capacity (*k*) and a significantly higher residual conversion at long cycle numbers (*X*_r_). This behavior is consistent with reduced sintering due to the lower calcination temperature enabled by the presence of H_2_.

Although no postcycling textural characterization was performed, the observed evolution of CO_2_ carrying capacity with the number of reaction cycles can be interpreted in terms of the well-known mechanism governing CaO-based sorbents. During carbonation, the formation of a CaCO_3_ product layer progressively limits diffusion and reduces the effective reactive surface area. Therefore, the conversion is closely related to the available surface and the thickness of the product layer formed on CaO particles that mainly depends on the carbonation temperature.^30^ Under the milder calcination temperatures enabled by DERWGS conditions, sintering is reduced, leading to better preservation of the pore structure and surface area over repeated cycles. This results in a slower decay of the CO_2_ carrying capacity compared to conventional CaL conditions.

To determine the effect of calcination temperature on the reaction rate under DERWGS conditions, a series of calcination experiments was carried out at temperatures between 600 and 800 °C in an atmosphere of 100 vol % H_2_. This temperature range was selected as it is favorable for maximizing CO production according to the combined equilibrium of CaCO_3_ decomposition and the reverse water–gas shift (RWGS) reaction (Figure 3).^15^ It should be noted that these temperatures are significantly lower than those typically employed in conventional calcination processes operated in air or in systems designed to generate a concentrated CO_2_ stream at the calciner outlet for subsequent utilization or storage.^10,11^ Figure 8 presents the CaCO_3_ conversion curves as a function of time obtained during the first (left) and fifth (right) calcination cycles. A strongly temperature-dependent behavior is observed over the entire range investigated. In particular, at the lowest temperatures tested, the conversion curves exhibit a pronounced sigmoidal shape, which is characteristic of reactions initiated after an induction period. Such behavior is commonly associated with the activation of specific reactive sites, potentially linked to crystal defects or the progressive formation of a reactive product phase.^23^ In the present system, CaCO_3_ decomposition may be initiated through the formation of CaO, which can subsequently participate in the RWGS reaction by catalyzing the hydrogenation of the CO_2_ released during calcination—a catalytic activity of CaO that has been previously reported in the literature.^15,16^ The resulting consumption of CO_2_ via RWGS shifts the calcination equilibrium, thereby promoting further carbonate decomposition.

**Figure 8:**
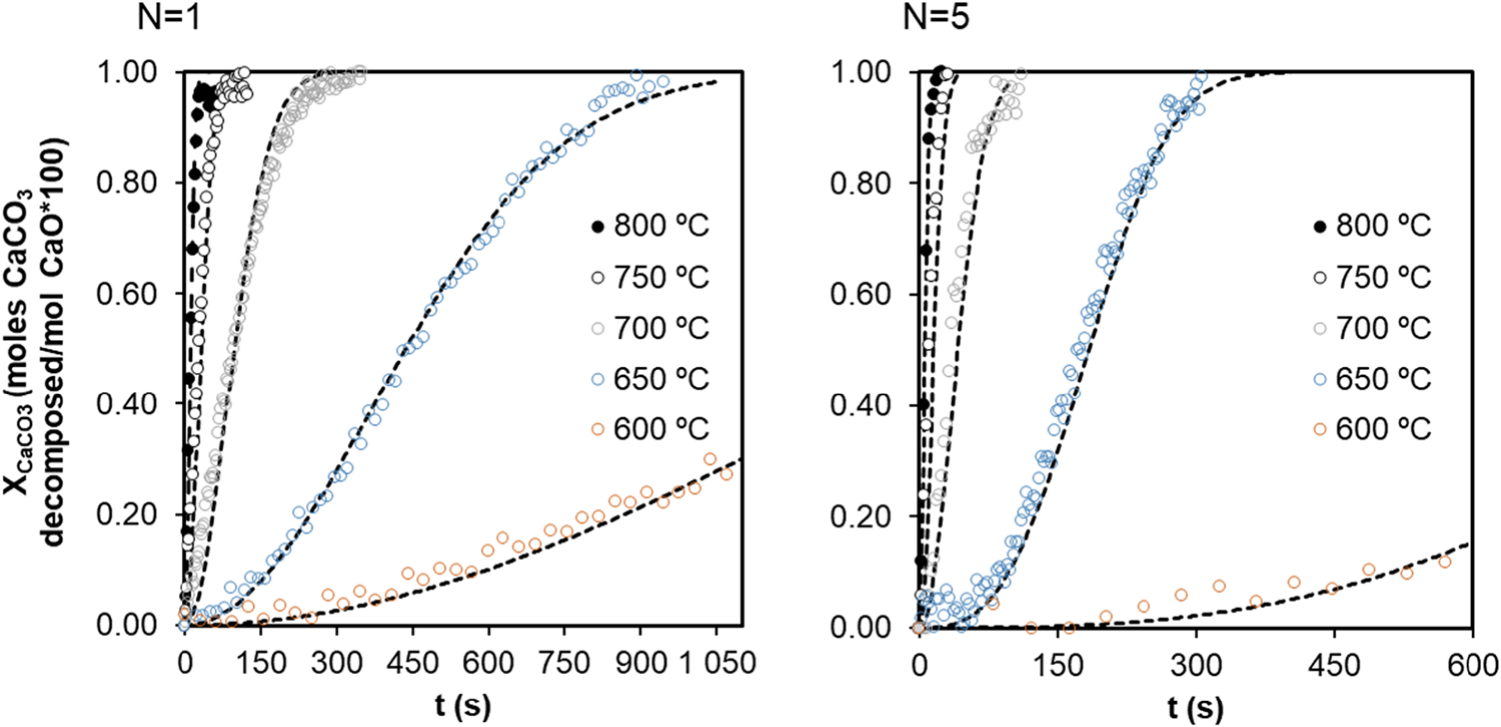
CaCO_3_ conversion curves vs time at different calcination temperatures. (Left) First calcination cycle. (Right) Fifth calcination, after four calcination/carbonation cycles. Symbols represent experimental data, and dashed lines correspond to the predictions from expression 6.

The sigmoidal character of the conversion curves becomes more pronounced at lower temperatures, where the equilibrium CO_2_ partial pressure under DERWGS conditions is particularly low. This observation is consistent with a mechanism in which the coupling between calcination and CO_2_ hydrogenation plays a dominant role in controlling the overall reaction rate. The strong temperature sensitivity evident in Figure 8 further suggests a relatively high apparent activation energy for the global DERWGS process. By comparison, reported activation energies for CaCO_3_ calcination under inert or vacuum conditions typically fall within the range 100–205 kJ mol^–1^.^18,19,22^

To elucidate the effect of CO_2_ in the reacting gas on CaCO_3_ calcination kinetics, additional experiments were performed at 780 and 750 °C with varying CO_2_ concentrations in the gas phase, expressed relative to the equilibrium CO_2_ partial pressure defined by eq ([Disp-formula eq4]). The resulting conversion curves are shown in Figure 9. As can be observed, even small additions of CO_2_ significantly inhibit the calcination rate. At 780 °C, a CO_2_ concentration of 5 vol % corresponds to approximately 0.3 p_CO_2_,eq_ (Figure 9, left), while at 750 °C the same concentration corresponds to around 0.6 p_CO_2_,eq_ (Figure 9, right). In both cases, the presence of CO_2_ markedly reduces the rate of CaCO_3_ conversion, with the inhibitory effect becoming increasingly pronounced as the equilibrium CO_2_ partial pressure is approached. This behavior is consistent with the thermodynamic and kinetic coupling inherent to the DERWGS system and highlights the necessity of explicitly accounting for the influence of CO_2_ on the reaction rate. Accordingly, the dependence of CaCO_3_ decomposition kinetics on CO_2_ concentration is incorporated into the kinetic expressions developed to describe conversion as a function of time.

**Figure 9:**
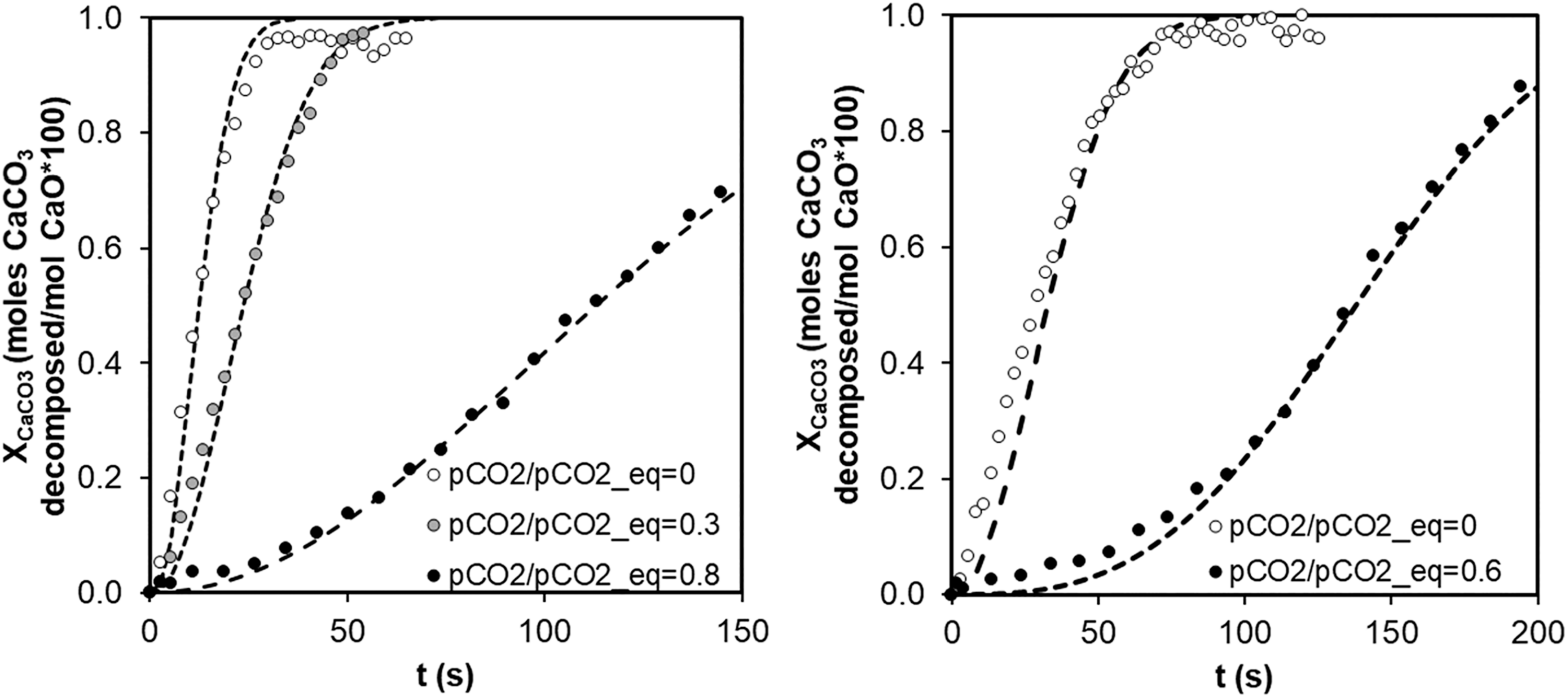
CaCO_3_ conversion curves vs time, during the first calcination cycle with different CO_2_ contents in the reaction atmosphere, referred to the equilibrium according to eq ([Disp-formula eq4]). (Left) At 780 °C. (Right) At 750 °C. Symbols represent experimental data, and dashed lines represent predictions from expression 6.

In view of the experimental results, CaCO_3_ conversion curves were analyzed using the Avrami–Erofeev model, which assumes random instantaneous nucleation followed by *n*-dimensional growth of the product phase.^31^ This formulation has been successfully applied to describe CaCO_3_ calcination under close-to-equilibrium conditions and in conventional calcium looping systems.^23,32^ As shown in Figure 10 (left), the characteristic reaction rate–conversion dependence predicted by the Avrami–Erofeev model is the only one among the tested formulations that reproduces the sigmoidal behavior observed experimentally in Figures 4, 8, 9, and 11, particularly at low temperatures. By contrast, the volumetric model (VM), grain model (GM), and random pore model (RPM) predict monotonically decreasing or only weakly inflected rate profiles, which are less consistent with the induction period and subsequent rate acceleration observed under DERWGS conditions. Accordingly, an overall homogeneous reaction pattern was assumed to describe CaCO_3_ conversion as a function of time and to determine the intrinsic kinetic parameters of the global reaction. This assumption is also consistent with previous studies reporting homogeneous conversion behavior for particle sizes below 300 μm.^22^ In the Avrami–Erofeev expression, the conversion-dependent term includes an exponent n, which was fixed at *n* = 2. This value is the characteristic of transient nucleation and two-dimensional growth, a mechanism previously identified for CaCO_3_ calcination by *in situ* XRD and later corroborated at the macroscopic scale by Li et al.^32^ Moreover, as illustrated in Figures 6 and 10 (left), the *n* = 2 formulation adequately captures the experimentally observed evolution of reaction rate with conversion. On this basis, the following kinetic expression was adopted to describe CaCO_3_ decomposition in hydrogen

6 $$\frac{dX_{CaCO3}}{dt}=\frac{k_{CaCO3}}{X_{carbN-1}}(n\left(1-X_{CaCO3}\right){\left(-\ln(1-X_{CaCO3}\right))}^{\left(1-1/n\right)})\left(1-\frac{pCO_{2}}{pCO_{2,eq}}\right)p_{H2}$$

where *X*_carb,N–1_, represents the initial carbonate content of the solid prior to calcination, calculated from eq ([Disp-formula eq5]). The kinetic expression includes (i) an Avrami–Erofeev conversion term, (ii) a thermodynamic driving-force term relative to the equilibrium CO_2_ partial pressure, as commonly used for calcination near equilibrium,^22,23^ and (iii) a first-order dependence on hydrogen partial pressure. The apparent kinetic constant k_CaCO3_ follows an Arrhenius-type dependence, defined by a pre-exponential factor *k*_0,CaCO3_ and an activation energy *E*_a,CaCO3_.To determine these kinetic parameters, the experimental values of d*X*_CaCO3_/dt were plotted against the corresponding conversion function *f*(*X*_CaCO3_) for all experiments performed at different temperatures and calcination cycles. The values of *k*_CaCO3_/*X*_carb,N–1_ were extracted from the maxima of these curves. The resulting kinetic constants were subsequently represented in Arrhenius form as a function of 1/*T*, as shown in Figure 10 (right). A highly linear relationship was obtained (*R* ^2^ = 0.993), confirming the suitability of the proposed kinetic formulation and the strong temperature dependence of the global DERWGS reaction. For this analysis, calcination cycles 1, 5, and 10 were considered, yielding three experimental points per temperature.

**Figure 10:**
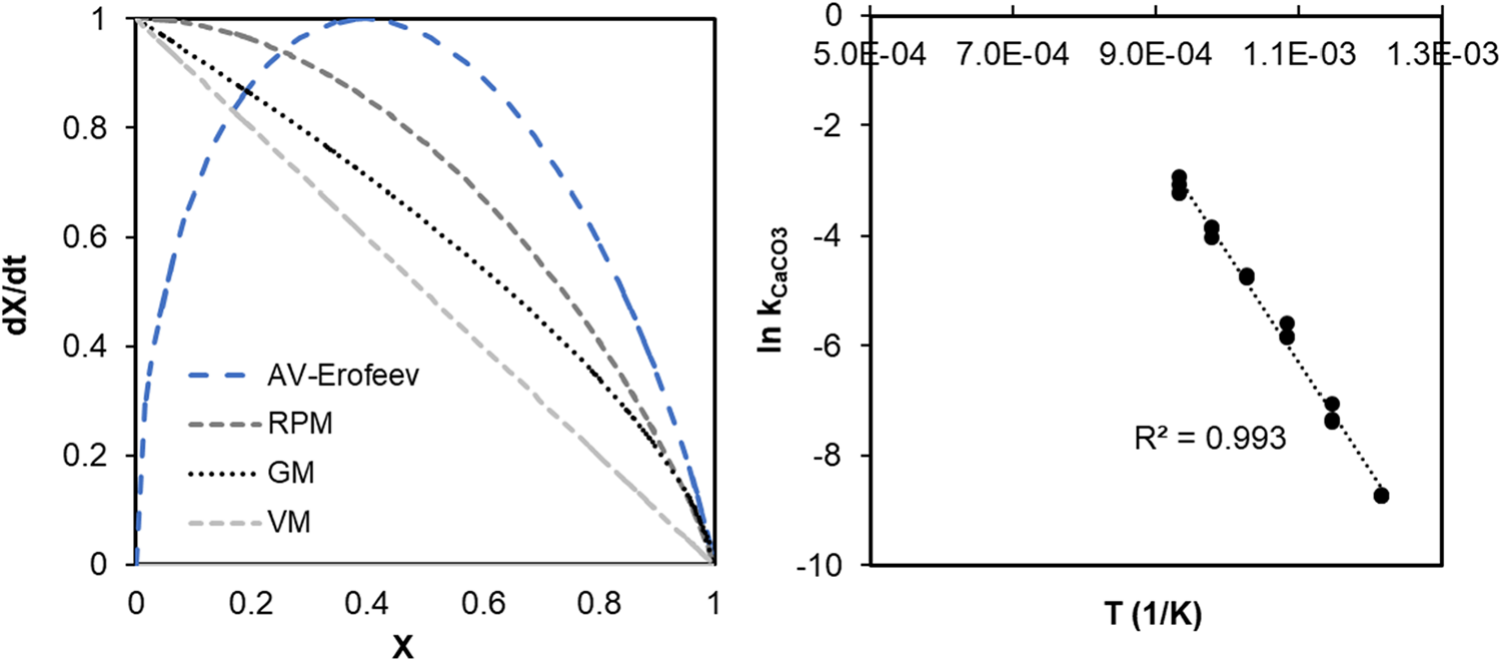
(Left) Determination of the kinetic models evaluated. (Right) Arrhenius plot of the individual kinetic constants *k*_CaCO3_ obtained for 1st, 5th, and 10th calcination cycles.

**Figure 11:**
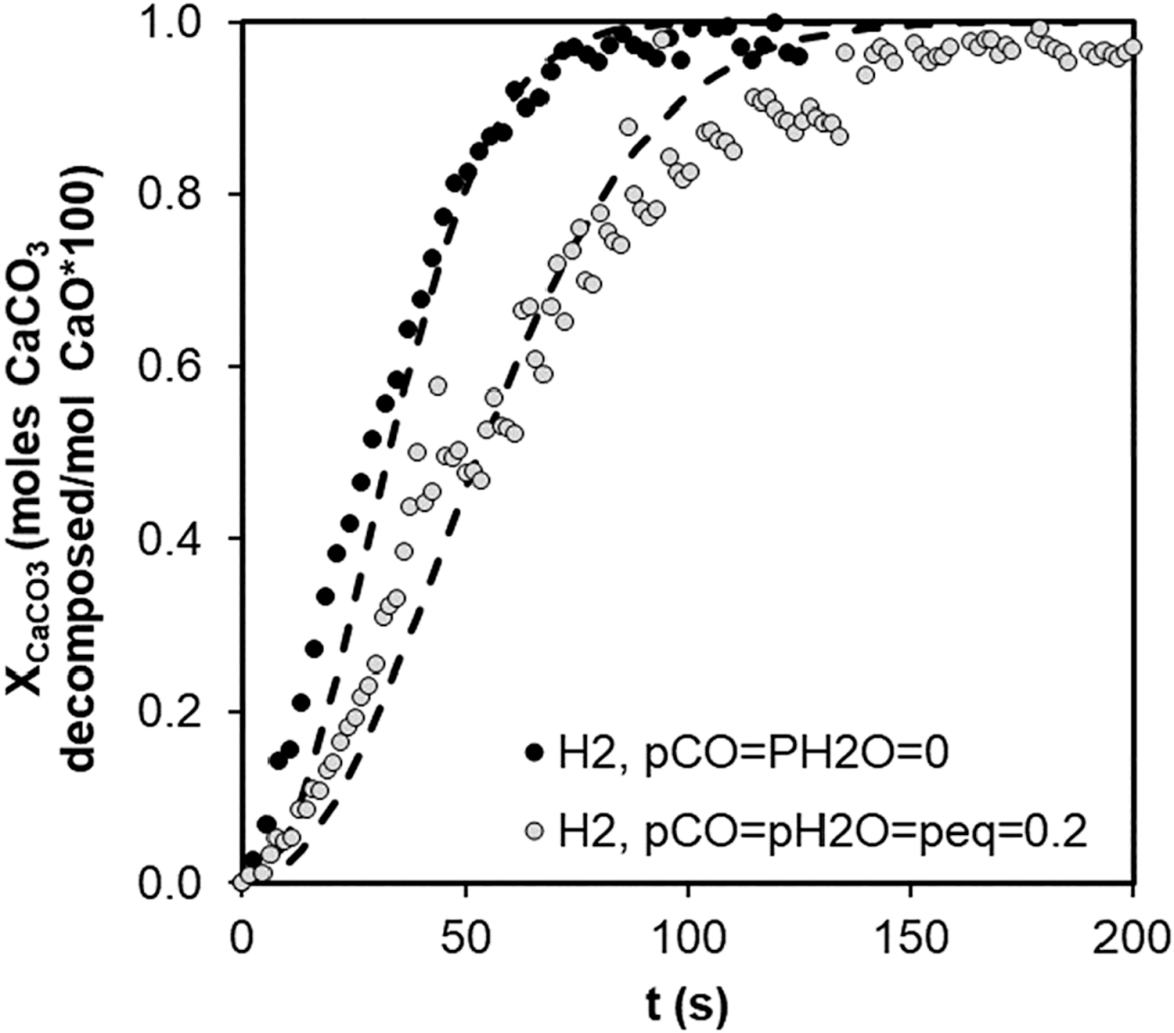
CaCO_3_ conversion curves vs time, during the first calcination cycle at 750 °C in the presence of CO and H_2_O partial pressure. Symbols represent experimental data, and dashed lines represent kinetic expression predictions.

The kinetic parameters obtained from this procedure are summarized in Table 3. The activation energy determined for CaCO_3_ decomposition in H_2_ was 184 kJ mol^–1^, indicating a highly temperature-sensitive process. This value is consistent with those reported in the literature for CaCO_3_ hydrogenation or reductive calcination under comparable conditions, namely 161.3 kJ mol^–1^ in a microfluidized bed reactor between 610 and 760 °C,^25^ and up to 236 kJ mol^–1^ when evaluated at significantly lower temperatures, starting from 430 °C.^9,14^ The value obtained here is also in good agreement with the activation energies predicted by Li and Cai^33^ from density functional theory calculations coupling surface reactions, structural transformations, and nucleation phenomena, which become particularly relevant under near-equilibrium calcination conditions.

**Table 3:** Parameters for the Kinetic Equation ([Disp-formula eq6])

*n* = 2
*k*_oCaCO3_ (s^–1^) = (6.36 ± 0.5) × 10^7^
*E*_aDERWGS_ (kJ mol^–1^) = 184 ± 4

The predictive capability of eq ([Disp-formula eq6]) is illustrated by the dashed lines in Figure 8, where the model satisfactorily reproduces the CaCO_3_ conversion profiles measured at different temperatures for solids with different initial carbonate contents, corresponding to the first and fifth calcination cycles. The relatively low uncertainty in the activation energy indicates a robust estimation, while the higher relative uncertainty in the pre-exponential factor reflects the known correlation between the Arrhenius parameters.

The applicability of the kinetic expression was further assessed under gas compositions containing reaction products from the DERWGGS process. As shown in Figure 9, the model accurately captures the inhibitory effect of CO_2_ over a wide range of *p*_CO2_/*p*_CO2,eq_ ratios, including values up to 0.8, confirming that the approach remains valid under close-to-equilibrium conditions.

Additional experiments were performed at 750 °C in the presence of CO and H_2_O at concentrations corresponding to DERWGS equilibrium conditions (*p*_CO_ = *p*_H2O_ = 0.2, *p*_H2_ = 0.6). The resulting conversion curves, shown in Figure 11, are also well described by eq ([Disp-formula eq6]). Importantly, the agreement between model and experiment indicates that neither CO nor H_2_O exerts an independent inhibiting or promoting effect on the intrinsic CaCO_3_ decomposition rate. Instead, the lower overall calcination rate observed relative to the case of pure H_2_ can be fully explained by the reduced hydrogen partial pressure during the experiment.

This observation provides important mechanistic insight. If hydrogen were directly attacking the CaCO_3_ lattice to produce CO and H_2_O in a single-step reductive decomposition pathway, the presence of reaction products such as CO and H_2_O would be expected to affect the forward reaction rate through product inhibition or reverse-reaction effects. In contrast, the absence of any measurable kinetic influence of CO and H_2_O on CaCO_3_ decomposition, together with the strong inhibitory role of CO_2_, supports a mechanism based on two consecutive reactions: first, conventional CaCO_3_ calcination to CaO and CO_2_; and second, rapid conversion of the released CO_2_ with H_2_ via the RWGS reaction. Under these conditions, the role of hydrogen is therefore not to directly reduce the carbonate lattice, but rather to consume the CO_2_ formed during calcination, thereby shifting the equilibrium and enhancing overall CaCO_3_ decomposition. Overall, the results from Figures 9 and 11 indicate that, in the presence of excess H_2_ and CaCO_3_, CO_2_ is the only gas-phase product that directly limits the calcination step through the equilibrium term in eq ([Disp-formula eq6]), whereas its subsequent consumption via RWGS promotes further decomposition. This behavior is fully consistent with the DERWGS concept as a coupled calcination–RWGS process and provides strong indirect support for a sequential reaction mechanism rather than direct hydrogenation of CaCO_3_.

## 4 Conclusions

This work provides a detailed kinetic investigation of CaCO_3_ decomposition in hydrogen under conditions relevant to the desorption-enhanced reverse water–gas shift (DERWGS) process, with the aim of establishing a robust kinetic basis for the design of both once-through low-carbon lime production systems and cyclic calcium looping (CaL)-based CO_2_ capture and utilization processes. Calcination in H_2_ was found to be markedly faster than under inert conditions, enabling complete CaCO_3_ decomposition at temperatures approximately 150 °C lower than those required in conventional calcination environments. This enhanced reaction rate has direct implications for process intensification and energy demand reduction in lime production and CaL regeneration stages. In addition, CaO produced under DERWGS-relevant conditions exhibited significantly improved cyclic performance, with nearly double the residual CO_2_ carrying capacity compared to sorbents calcined under standard CaL conditions, indicating reduced sintering and slower sorbent deactivation, mainly due to milder calcination temperatures enabled by the presence of H_2_.

For particle sizes below 100–200 μm, CaCO_3_ decomposition proceeded in a homogeneous reaction regime, with the overall calcination time primarily governed by the initial carbonate content of the solid. The conversion–time behavior exhibited a pronounced sigmoidal shape, particularly at lower temperatures, revealing that the reaction kinetics deviate from classical monotonic shrinking-core or volumetric descriptions. Among the kinetic models evaluated, the Avrami–Erofeev expression with an exponent *n* = 2 provided the best representation of the experimental rate–conversion trends, consistent with a mechanism involving transient nucleation and two-dimensional growth of the CaO phase. A kinetic formulation combining this conversion term with an Arrhenius-type temperature dependence, a first-order dependence on hydrogen partial pressure, and a thermodynamic driving-force term with respect to the equilibrium CO_2_ partial pressure successfully reproduced experimental conversion curves over a wide range of temperatures, calcination cycles, and gas-phase compositions. The apparent activation energy obtained for CaCO_3_ decomposition in H_2_ (184 ± 4 kJ mol^–1^) confirms the strong temperature sensitivity of the global DERWGS process and agrees well with values reported for reductive calcination and hydrogen-assisted CaCO_3_ decomposition in the literature. Gas-phase effects were shown to play a critical role in controlling the reaction rate. CO_2_ was identified as the only species exerting a direct inhibiting effect on CaCO_3_ decomposition, with the rate decreasing sharply as the equilibrium CO_2_ partial pressure was approached. In contrast, CO and H_2_O did not produce any measurable additional influence on the intrinsic calcination kinetics beyond the effect associated with reduced hydrogen partial pressure. These observations provide strong mechanistic evidence that CaCO_3_ decomposition under DERWGS conditions proceeds via a sequential pathway, consisting of conventional calcination to CaO and CO_2_ followed by rapid hydrogenation of the released CO_2_ via the RWGS reaction. Direct reductive cleavage of the carbonate lattice by hydrogen is therefore not supported under the conditions investigated.

Overall, the kinetic framework developed in this work offers a physically consistent and practically relevant basis for modeling, reactor design, and scale-up of DERWGS systems. The results reinforce the potential of hydrogen-assisted calcination as an intensified route for simultaneously enabling low-carbon lime production and CO_2_ utilization, while maintaining compatibility with existing CaL-based industrial infrastructures.
